# A national framework for strengthening public health laboratory preparedness and resilience in Jordan: advancing health security, One Health, and early warning capacity

**DOI:** 10.3389/fpubh.2026.1962613

**Published:** 2026-10-20

**Authors:** Dalia Kashef Zayed, Tasnim Abu Al Khair, Ruba A. Al-Smadi, Mohammad H. Gharaibeh, Tareq L. Mukattash, Adel Belbiesi, Ala'a B. Al-Tammemi

**Affiliations:** 1 Jordan Center for Disease Control, Amman, Jordan; 2 Department of Health Economics and Healthcare Administration, The Institute of Public Health, The University of Jordan, Amman, Jordan; 3 Princess Iman Center for Research and Laboratory Sciences, Royal Medical Services, Amman, Jordan; 4 Department of Basic Veterinary Medical Sciences, Jordan University of Science and Technology, Irbid, Jordan; 5 Department of Clinical Pharmacy, Faculty of Pharmacy, Jordan University of Science and Technology, Irbid, Jordan

**Keywords:** early warning, epidemic intelligence, health security, laboratory governance, laboratory systems strengthening, One Health, public health laboratories, quality management systems

## Abstract

National laboratory systems are central to epidemic intelligence, early detection, and implementation of the International Health Regulations. In Jordan, substantial diagnostic capacity exists, but laboratory functions remain distributed across institutions and sectors, with persistent challenges in governance, quality systems, biosafety and biosecurity, workforce capacity, referral arrangements, and data interoperability. In 2023, Jordan undertook a national laboratory mapping using a multi-method approach comprising review of 226 national and international documents, 20 key-informant interviews, and 23 field visits covering 27 laboratories in four governorates. Findings were analyzed thematically using the LABNET framework and reviewed through a national multisector stakeholder validation process. Building on these findings and the subsequent endorsement of Jordan's National One Health Laboratory Strategic Plan 2026–2030, we propose an implementation-oriented framework for strengthening the national laboratory system. The framework is organized around five interdependent pillars: governance, legislation, and strategic direction; a coordinated national laboratory network; quality management, external quality assessment, and accreditation pathways; workforce competency development and retention; and digital transformation with multisector One Health information exchange. Rather than establishing new parallel structures, the framework is intended to connect and strengthen existing capacities while preserving the statutory and technical mandates of participating sectors. A phased roadmap for 2026–2030 translates these pillars into operational priorities and links them to cross-cutting enablers, including sustainable financing, biosafety and biosecurity, cybersecurity, equity, emergency preparedness, and monitoring, evaluation, and learning. The framework is prospective and has not yet been evaluated as an integrated intervention. Its value will therefore depend on implementation fidelity and prospective assessment against defined baselines. If effectively operationalized, it could strengthen referral performance, diagnostic reliability, reporting timeliness, multisector risk assessment, and the use of laboratory intelligence for early warning and coordinated response. Although developed for Jordan, the framework offers a context-adaptable model for countries seeking to strengthen laboratory resilience through phased, multisectoral, and One Health–oriented reform.

## 1 Introduction

A resilient national laboratory system is a cornerstone of health security and a core capacity under the International Health Regulations (IHR 2005) (1). Laboratories provide the foundation for early detection of priority pathogens, outbreak confirmation, antimicrobial resistance (AMR) surveillance, and One Health risk assessment functions that are essential for preventing, controlling, and responding to public health threats (1–3). High quality pathology and laboratory diagnostic services are fundamental to improving global health outcomes, as effective disease prevention, treatment, and control depend on timely and accurate diagnosis (4). Access to reliable diagnostics is therefore a critical prerequisite for achieving better health outcomes. However, substantial diagnostic gaps persist worldwide; the Lancet Commission on Diagnostics estimated that approximately 47% of the global population has little or no access to essential diagnostic services (5).

The COVID-19 pandemic reaffirmed the centrality of laboratory networks to national preparedness, exposing persistent global challenges, including fragmented governance, weak quality systems, and limited multisector data interoperability, particularly across low- and middle-income countries (LMICs) (6, 7).

Regional evidence underscores how uneven this foundation remains. In a 2022 WHO Eastern Mediterranean Region (EMR) scorecard covering 21 Member States, only four (19%) had fully implemented a national laboratory policy and five (24%) an integrated national laboratory strategic plan; 16 (76%) had not fully actualized either instrument (7). Jordan's own trajectory reflects these regional constraints.

Findings from Jordan's 2026 State Party Self-Assessment Annual Report (SPAR) support the presence of strong diagnostic capacity in selected laboratories, with laboratory diagnostic capacity for priority pathogens rated among the country's strongest domains (8). Although the broader laboratory system is distributed across multiple ministries and sectors, this multisectoral landscape presents a valuable opportunity to establish a comprehensive, One Health–oriented national laboratory network. At the same time, it poses important coordination challenges, including variability in quality standards, uneven biosafety and biosecurity practices, and inconsistent data sharing and reporting mechanisms across sectors (9, 10).

In 2023, Jordan conducted its first integrated national mapping of laboratory capacities for communicable diseases, which, to our knowledge, represents the most comprehensive diagnostic systems assessment conducted in Jordan to date (9). The mapping used a multi-method approach combining a desktop review of 226 national and international documents, 20 key-informant interviews with stakeholders from public, private, military, veterinary, and academic sectors, and 23 field visits covering 27 laboratories across four governorates. Evidence was analyzed thematically and synthesized using the LABNET framework to identify recurrent strengths and system-level gaps across core laboratory capacities, with findings reviewed through a national multisector stakeholder validation process. The assessment identified important strengths, including a skilled workforce and technically capable facilities, alongside recurrent constraints related to fragmented governance and coordination, variable implementation of quality systems and biosafety-biosecurity requirements, workforce capacity, and limited digital and cross-sector data integration (9, 10). These challenges are consistent with those documented in the WHO EMR's Regional Laboratory Strengthening Strategy and the Global Health Security Agenda (GHSA) action packages on laboratory systems and biosafety-biosecurity (2, 11).

For this policy perspective, the published mapping findings constitute the empirical starting point rather than a new analysis of the primary mapping data. Recurrent gaps were consolidated into five interrelated policy domains: (i) governance, legislation, and strategic direction; (ii) national laboratory networking and multisector coordination; (iii) quality systems, External Quality Assessment (EQA), and accreditation; (iv) workforce capacity and competency development; and (v) digital transformation, data governance, and One Health integration. Proposed actions within these domains were then contextualized against relevant normative frameworks, including the WHO's Laboratory Quality Stepwise Implementation (LQSI) tool, ISO 15189:2022 for medical laboratory accreditation, and the Quadripartite One Health Joint Plan of Action (3, 12, 13). However, translating these frameworks into coherent national policy requires political commitment, clear governance arrangements, sustainable financing, and phased implementation tailored to country context.

Since the mapping, Jordan has endorsed the National One Health Laboratory Strategic Plan (2026–2030) (14), the framework presented here is therefore not a parallel national strategy; it is an evidence-informed, implementation-oriented structure intended to complement the endorsed plan by linking mapping-derived gaps to coordinated actions, a phased roadmap, and measurable indicators.

## 2 A national framework for laboratory system strengthening (2026–2030)

Jordan's national mapping identified system strengths alongside gaps in governance, quality systems, biosafety-biosecurity, workforce capacity, and cross-sector data integration (9). The five pillars below are policy recommendations derived from those documented gaps and contextualized against national, regional, and international frameworks. Accordingly, assessment findings are described as findings of the mapping, whereas proposed institutional arrangements and strengthening actions are explicitly presented as author recommendations. Unless otherwise supported by a formal mandate, institutional roles described within the framework should be interpreted as proposed complementary governance arrangements. Figure 1 summarizes the five pillars, expected implementation outputs, and anticipated longer-term impacts.

**Figure 1:**
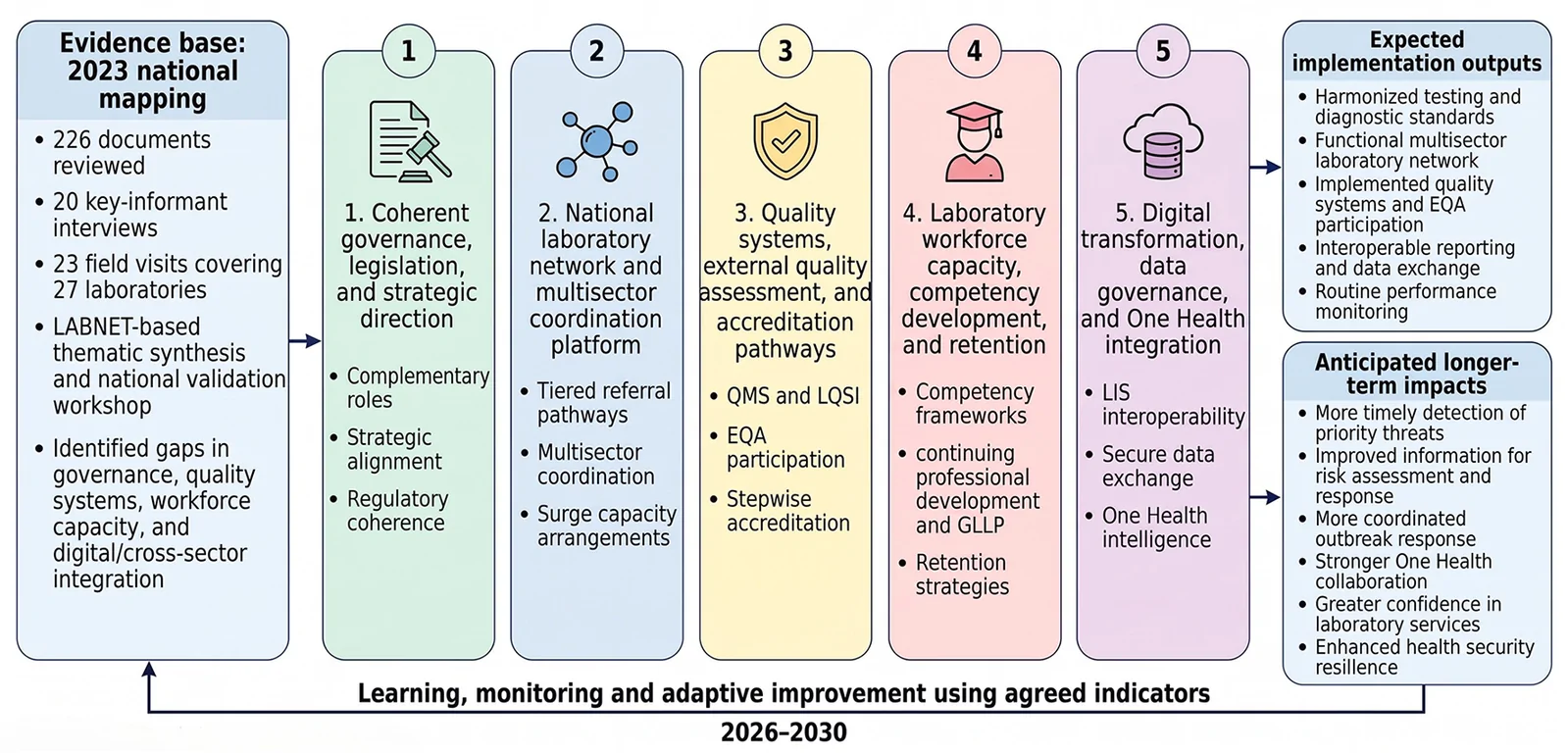
Five-pillar national framework for laboratory system strengthening in Jordan, 2026−2030. Outputs and impacts are prospective and require evaluation. QMS, quality management system; EQA, external quality assessment; LIS, laboratory information systems; GLLP, Global Laboratory Leadership Program; LQSI, Laboratory Quality Stepwise Implementation tool.

### 2.1 Pillar 1: coherent governance, legislation, and strategic direction

The aforementioned 2023 mapping identified fragmented governance and the absence, at the time of assessment, of a unified national laboratory strategic framework as important system constraints (9). Since then, Jordan has endorsed the National One Health Laboratory Strategic Plan (2026–2030) (14). The policy priority is therefore to operationalize the endorsed plan through costed actions, clear accountability, coordinated governance, and targeted review of laboratory, licensing, biosafety-biosecurity, and data-governance instruments. Building on existing institutional responsibilities, this pillar proposes a complementary governance arrangement in which the Ministry of Health, through the Central Public Health Laboratory (CPHL), would provide technical leadership for laboratory standards, diagnostic algorithms, quality requirements, and biosafety-biosecurity guidance within the national network. The Jordan Center for Disease Control (JCDC), consistent with its national coordination and preparedness functions, would support multisector coordination and integration of laboratory intelligence into surveillance, early warning, and preparedness mechanisms. These proposed functions are intended to clarify interfaces and strengthen coordination rather than replace or duplicate statutory mandates.

The WHO EMR guidance supports national laboratory leadership at ministerial level, linked to central public health laboratories and supported by multisectoral coordination mechanisms (2, 7). The precise institutional configuration in Jordan should remain consistent with national legislation and the governance arrangements established under the endorsed strategic plan.

### 2.2 Pillar 2: a national laboratory network and multisector coordination platform

Jordan's laboratory landscape comprises human health, animal health, environmental health, and food safety laboratories distributed across public, private, military, and academic institutions (9, 10). Here, we propose a formal National Laboratory Network, supported by a multisector steering mechanism, to connect these capacities through defined referral pathways, communication channels, and emergency arrangements, consistent with WHO EMR and GHSA guidance (2, 11). One Health integration within the network should be functional rather than structural, allowing participating sectors to retain their statutory and technical mandates while contributing complementary intelligence to a shared surveillance and response architecture. Human health laboratories would provide diagnostic and surveillance data on priority communicable diseases; veterinary laboratories would contribute information on animal health and zoonotic pathogens; food safety laboratories would generate evidence on foodborne hazards and outbreaks; and environmental laboratories would monitor relevant environmental exposures and pathogens. Plant health laboratories should be engaged where plant-associated hazards or food-system risks intersect with priority One Health threats. For hazards that span sectors, predefined protocols should support timely cross-sector notification, referral or sharing of relevant specimens and isolates where appropriate, exchange of laboratory and epidemiological data, joint risk assessment, and coordinated outbreak investigation and response (3, 15, 16).

A tiered network with defined central, regional, and peripheral functions should also specify specimen-referral routes, transport requirements, surge-testing arrangements, and escalation triggers. Scheduled multisector meetings, simulation exercises, after-action reviews, and joint planning can test these arrangements and address the fragmented surveillance and cross-sector coordination barriers described regionally and in Jordan (9, 10, 17).

### 2.3 Pillar 3: quality systems, external quality assessment, and accreditation pathways

The mapping identified substantial variation in implementation of quality management systems (QMSs), EQA, and corrective action processes across laboratories (9). The WHO's LQSI tool provides a structured pathway for progressive QMS adoption, while ISO 15189:2022 defines requirements for quality and competence in medical laboratories (12, 13). Experience with transition to ISO 15189:2022 further emphasizes early gap analysis, risk-based thinking, and sustained staff engagement, although published reports remain context-specific (18).

To address these gaps, we propose coordinated national EQA arrangements with transparent participation, feedback, corrective action, and performance review processes. Priority reference laboratories could progress through phased QMS milestones toward ISO 15189 accreditation, while broader network laboratories receive stepwise quality improvement support. This approach would extend reliable quality practices without implying that accreditation is immediately feasible or necessary for every laboratory (12, 13, 19–21).

### 2.4 Pillar 4: laboratory workforce capacity, competency development, and retention

A capable and equitably distributed workforce is fundamental to system resilience. Regional data underline the scale of this gap: in a 2022 WHO EMR scorecard of 21 Member States, only six (29%) demonstrated capacity in laboratory human resources for service delivery, four (19%) licensed laboratory workers on the basis of education, training and competency assessment, and no low- or lower-middle-income Member State had implemented a national laboratory staffing plan based on workload forecasting (7).

The Global Laboratory Leadership Program (GLLP) provides a competency framework spanning laboratory systems, leadership, management, communication, QMS, biosafety and biosecurity, disease surveillance and outbreak investigation, emergency preparedness and response, and research (22, 23). Building on this framework, and in alignment with the WHO guidance on human resources for health (24), we propose a national laboratory competency framework that defines expectations for priority functions across sectors and tiers, including diagnostic practice, biosafety-biosecurity, quality, digital literacy, leadership, and emergency response.

Structured continuing professional development, mentorship, and partnerships with academic and professional institutions should accompany competency assessment. Workforce planning should also address distribution, succession, and retention in underserved areas and specialized roles. Evidence supporting rural recruitment and retention strategies derives mainly from broader health worker cadres; application to laboratory professionals should therefore be adapted and evaluated in Jordan rather than assumed to transfer directly (25).

### 2.5 Pillar 5: digital transformation, data governance, and one health integration

The mapping identified limited interoperability between laboratory information systems (LISs) and surveillance platforms as a major constraint to timely information flow (9). We propose a national minimum laboratory dataset, agreed reporting triggers, interoperable exchange standards, and governance arrangements linking laboratory outputs with surveillance and early warning systems, consistent with the WHO digital health guidance (26, 27).

Interoperability should extend beyond human-health systems to enable standardized, secure exchange of relevant laboratory and surveillance information across human health, animal health, food safety, environmental health and, where applicable, plant health. Data-sharing agreements, privacy and access controls, cybersecurity safeguards, common identifiers where feasible, and agreed minimum datasets should support joint risk assessment, coordinated outbreak investigation, and multisector analyses for priority One Health threats (3, 15, 16). These mechanisms position laboratories as contributors to multisector epidemic intelligence while preserving sector-specific responsibilities.

Together, the five pillars form a mutually reinforcing implementation package: governance enables coordination; network arrangements connect sectoral capacities; quality and workforce measures strengthen technical reliability; and digital and One Health integration convert laboratory outputs into actionable intelligence. The framework is intended to complement the National One Health Laboratory Strategic Plan (2026–2030), not replace it, and to provide a structure for sequencing implementation and evaluation.

## 3 Roadmap for implementation (2026–2030)

Operationalizing the framework requires a phased and adaptive roadmap aligned with Jordan's current policy context. The roadmap is presented for 2026–2030 and builds on foundations already established through the 2023 mapping and subsequent endorsement of the National One Health Laboratory Strategic Plan (2026–2030) (9, 14). The proposed three-phase roadmap is summarized in Figure 2, with detailed actions for each phase and pillar provided in Supplementary Table 1. The phases proposed provide a forward-looking sequence for translating existing strategic commitments into integrated laboratory-system functions.

**Figure 2:**
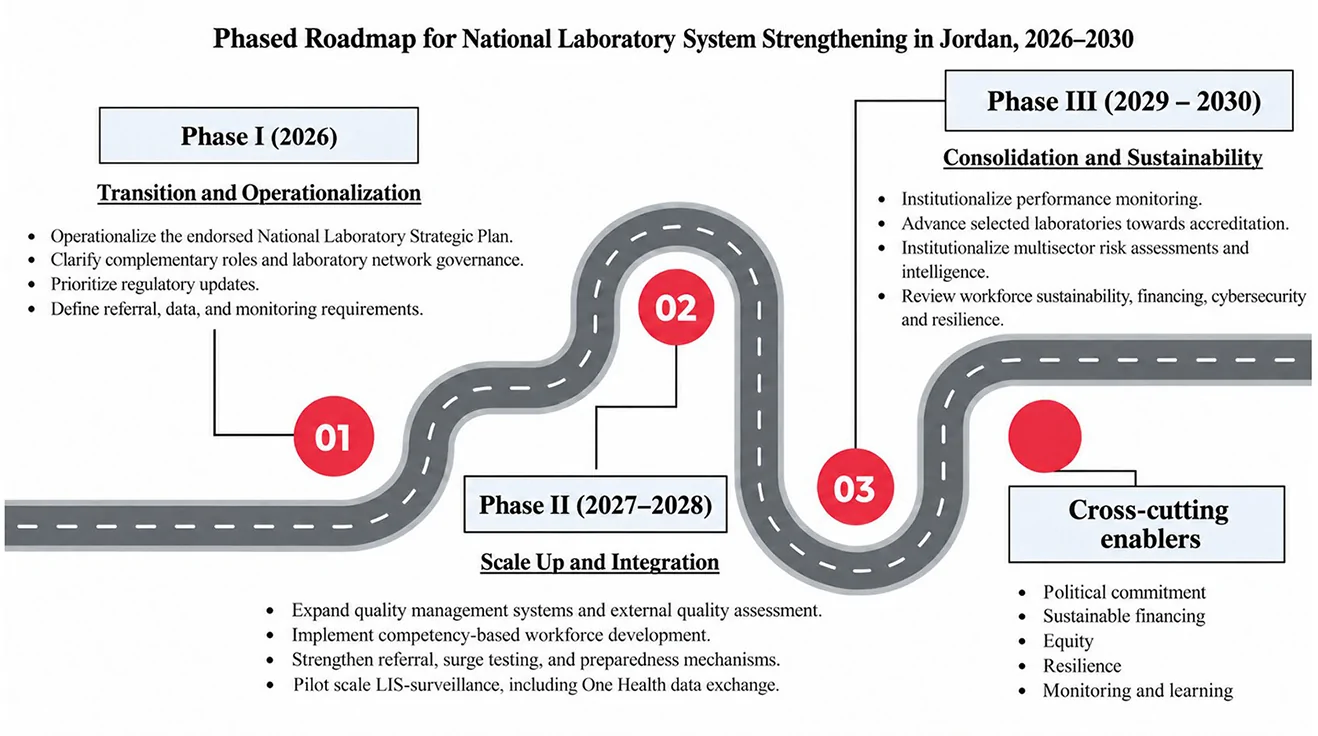
Three-phase roadmap for national laboratory system strengthening, 2026–2030.

Phase I - Transition and operationalization (2026) focuses on translating strategic commitments and mapping findings into implementable arrangements. Priorities include costing and operational planning for the endorsed National One Health Laboratory Strategic Plan, clarifying complementary institutional roles and coordination arrangements, operationalizing National Laboratory Network governance, prioritizing regulatory updates, and defining minimum data, referral, and monitoring requirements.

Phase II - Scale-up and integration (2027–2028) expands QMS and EQA across priority laboratories, implements competency-based workforce development, strengthens referral and surge-testing arrangements, and tests network readiness through exercises and after-action reviews. LIS-surveillance interoperability and secure multisector One Health information exchange should be piloted and progressively scaled for selected priority hazards.

Phase III - Consolidation and sustainability (2029–2030) embeds routine performance monitoring, extends QMS/EQA coverage, advances selected reference laboratories toward ISO 15189 accreditation, and institutionalizes interoperable reporting, joint risk assessment, and multisector intelligence products. Governance, financing, cybersecurity, workforce sustainability, and continuous learning should be periodically reviewed to maintain resilience and adapt to emerging threats.

Together, the roadmap underscores that laboratory system strengthening is not linear but iterative, requiring sustained political commitment, cross-sector coordination, and phased investments to translate technical capacity into actionable public-health intelligence.

## 4 Critical enablers for implementation

Implementing the framework requires cross-cutting enablers that determine feasibility and sustainability. Political commitment and governance continuity are central because laboratory strengthening spans institutions and sectors. The endorsed National One Health Laboratory Strategic Plan should remain the national strategic instrument, with implementation linked to clear accountability, national health-security and One Health governance, and a costed operational plan (14). Sustainable financing is equally important. Quality systems, workforce development, referral networks, biosafety/biosecurity, and digital infrastructure require predictable multi-year resources; financing should therefore be integrated into government planning and coordinated partner investment rather than rely on fragmented projects (24, 25, 27–29).

Equity and access should be monitored across governorates and sectors. Tiered referral arrangements, workforce planning, and digital connectivity can reduce geographic gaps, but implementation should be based on measured service needs and network performance. Private and other non-governmental laboratories should be incorporated through applicable reporting requirements, agreed referral pathways, and secure data-exchange mechanisms consistent with national regulation (1, 5, 9, 10, 25).

Additionally, risk mitigation and system resilience must be integrated into implementation. Biosafety and biosecurity standards, cybersecurity protocols, supply-chain redundancy, and surge-testing arrangements are critical to maintaining continuity of services during outbreaks, disasters, and crises. Alignment with the WHO biosafety guidance and the One Health Joint Plan of Action supports a risk-based approach across sectors (3, 20).

Finally, monitoring, evaluation, and learning should begin during operationalization rather than wait until the final phase. A concise core set of indicators should track governance functionality, network coverage and referral performance, EQA participation and QMS/accreditation progress, workforce competency, reporting timeliness and completeness, LIS interoperability, and multisector One Health investigations or risk assessments. Baselines, targets, data sources, reporting frequency, and accountable institutions should be agreed during operational planning rather than assigning arbitrary targets before standardized baseline measurement. Detailed illustrative indicators are presented in Supplementary Table 1. Cross-cutting enablers are summarized in Supplementary Figure 3.

Our proposed framework is prospective and has not been evaluated as an integrated intervention; its impacts are therefore anticipated, not demonstrated. If implemented effectively, stronger governance, network coordination, QMS/EQA, workforce competency, and interoperable multisector information exchange could improve referral performance, result reliability, reporting timeliness, joint risk assessment, and early warning. Prospective monitoring against defined baselines is required to determine whether these improvements translate into earlier detection and more coordinated response. Also, the framework reflects Jordan's multisector laboratory landscape. The five pillars may be adaptable elsewhere, but configuration and sequencing should reflect baseline capacity, resources, health system organization, and national priorities. Lower resource settings may need to prioritize essential diagnostic and referral capacity, foundational quality systems, workforce competencies, and feasible reporting before broader accreditation or advanced digital interoperability. The framework is therefore context-adaptable rather than directly replicable.

## 5 Conclusion and call to action

Strengthening Jordan's national laboratory system is central to health security, early detection, and IHR (2005) core capacities. The 2023 mapping provides an empirical basis for coordinated reform, while the subsequent National One Health Laboratory Strategic Plan (2026–2030) establishes the nationally endorsed strategic direction. The five-pillar framework presented here complements that plan by organizing system gaps and implementation actions into a sequenced, measurable pathway. Immediate priorities are to translate the endorsed plan into a costed operational program; clarify and, where appropriate, formalize complementary roles among Ministry of Health/CPHL, JCDC, and other participating authorities; operationalize National Laboratory Network governance and referral arrangements; and establish baselines and monitoring responsibilities. Under the governance model proposed in our policy perspective, Ministry of Health/CPHL could provide technical leadership for standards and quality, while JCDC could support multisector coordination and integration of laboratory intelligence into surveillance and preparedness, subject to national legal and governance instruments.

International and national partners, academic institutions, professional bodies, and private sector laboratories can support implementation where their investments align with national priorities, financing arrangements, and accountability mechanisms. Such support should strengthen national ownership rather than create parallel systems. The framework's effectiveness remains to be demonstrated through implementation, monitoring, and iterative learning. If effective, it could strengthen laboratory contributions to surveillance, One Health intelligence, preparedness, and national health security while offering adaptable system-strengthening domains for other contexts.

## Data Availability

The original contributions presented in the study are included in the article/Supplementary material, further inquiries can be directed to the corresponding author.
