# Supplementary material for: A national framework for strengthening public health laboratory preparedness and resilience in Jordan: advancing health security, One Health, and early warning capacity
Source: Front Public Health. 2026 Oct 20;14:1962613. doi: 10.3389/fpubh.2026.1962613 (PMC13727746; doi:10.3389/fpubh.2026.1962613)
Supplement: Supplementary file 1 [file Supplementary_file_1.docx]

**Supplementary Figure 3.** Cross-cutting enablers for implementation of the national laboratory strengthening framework.
