# Supplementary material for: A national framework for strengthening public health laboratory preparedness and resilience in Jordan: advancing health security, One Health, and early warning capacity
Source: Front Public Health. 2026 Oct 20;14:1962613. doi: 10.3389/fpubh.2026.1962613 (PMC13727746; doi:10.3389/fpubh.2026.1962613)
Supplement: Supplementary file 2 [file Table_1.docx]

**Supplementary Table 1**

**Phased Roadmap for National Laboratory System Strengthening in Jordan (2026–2030)**

| **Phase** | **Pillar 1 – Coherent governance, legislation, and strategic direction** | **Pillar 2 – A national laboratory network and multisector coordination platform** | **Pillar 3 – Quality systems, external quality assessment, and accreditation pathways** | **Pillar 4 – Laboratory workforce capacity, competency development, and retention** | **Pillar 5 – Digital transformation, data governance, and One Health integration** |
| --- | --- | --- | --- | --- | --- |
| **Phase I – Transition and operationalization (2026)** | • Operationalize and cost the endorsed National One Health Laboratory Strategic Plan (2026–2030).  • Clarify and, where appropriate, formalize complementary roles and coordination arrangements among MoH/CPHL, JCDC, and other relevant sectors.  • Prioritize review of laws, bylaws and regulations related to laboratories, biosafety/biosecurity, and data governance. | • Operationalize National Laboratory Network governance with representation from human health, animal health, food-safety, environmental and, where relevant, plant-health sectors.  • Validate tiered network and specimen-referral pathways for priority hazards.  • Link network governance to national health-security and One Health coordination mechanisms. | • Adopt stepwise QMS improvement using WHO LQSI or equivalent nationally agreed tools.  • Define coordinated EQA participation, feedback and corrective action arrangements.  • Harmonize priority SOPs and quality monitoring tools. | • Develop a national laboratory competency framework across priority functions and sectors.  • Establish baseline competency and workforce-distribution measures.  • Define CPD, mentorship and workforce-planning mechanisms. | • Define a national minimum laboratory dataset and priority reporting triggers.  • Agree interoperability requirements between LISs and surveillance platforms.  • Establish data-governance principles, secure data-sharing arrangements and baseline monitoring indicators. |
| **Phase II – Scale-up and integration (2027–2028)** | • Finalize and implement revised laboratory-related regulations, including licensing and biosafety/biosecurity provisions.  • Institutionalize annual planning and review cycles for the National One Health Laboratory Strategic Plan at national and sub-national levels.  • Align laboratory governance with broader health-sector and One Health strategies. | • Operationalize the National Laboratory Network through regular meetings, joint planning and defined terms of reference.  • Implement network-wide referral algorithms and surge-testing arrangements for outbreaks and public-health events.  • Conduct multisector simulation exercises and after-action reviews focusing on laboratory–surveillance linkages. | • Roll out LQSI-based QMS implementation in prioritized public health and reference laboratories.  • Expand EQA participation across sectors; institutionalize feedback, corrective actions and performance monitoring.  • Identify and prepare priority laboratories for ISO 15189 (medical) or ISO/IEC 17025 (veterinary, food-safety, environmental) accreditation through stepwise QMS improvement. | • Implement the competency framework through modular training, on-the-job coaching and leadership development programs.  • Establish career pathways and retention mechanisms, particularly for underserved governorates and critical technical roles.  • Integrate laboratory workforce planning into national human resources for health (HRH) processes. | • Pilot LIS–surveillance interoperability for selected priority diseases in chosen governorates.  • Implement secure digital reporting workflows from laboratories to surveillance and early-warning systems.  • Initiate multisector data-sharing and joint analyses across relevant human-health, animal-health, food-safety and environmental systems, with plant-health participation where relevant to prioritized One Health hazards. |
| **Phase III – Consolidation and sustainability (2029–2030)** | • Embed the National One Health Laboratory Strategic Plan within medium-term national health and One Health strategies, including financing frameworks.  • Institutionalize routine monitoring and evaluation using agreed indicators, baselines and targets at national and sub-national levels.  • Institutionalize governance reviews to address emerging threats and technological changes. | • Fully integrate the National Laboratory Network into national emergency preparedness and response structures.  • Formalize cross-border collaboration and information-sharing arrangements with regional partners.  • Maintain regular joint risk assessments and coordination meetings for priority threats. | • Advance selected national and regional reference laboratories toward ISO 15189 or ISO/IEC 17025 accreditation, as appropriate to their scope, and use them as centers of excellence for mentorship and training.  • Extend QMS and EQA coverage to additional laboratories based on risk and service priorities.  • Institutionalize continuous quality improvement cycles linked to national accreditation and licensing mechanisms. | • Consolidate a sustainable national laboratory workforce strategy, including funding lines for CPD, leadership development and retention.  • Evaluate impact of training and workforce reforms on service quality and readiness.  • Align national laboratory workforce initiatives with regional and global capacity-building programs. | • Fully integrate laboratory data into national surveillance, early-warning and One Health intelligence platforms.  • Operationalize joint multisector dashboards for zoonotic, AMR and climate-sensitive threats.  • Institutionalize cybersecurity, data protection and interoperability standards, with periodic review and adaptation to evolving risks. |

**Proposed core monitoring indicators**

| **Pillar** | **Illustrative indicators (baselines and targets to be nationally agreed)** |
| --- | --- |
| **Pillar 1 – Coherent governance, legislation, and strategic direction** | Governance mechanism functional; proportion of agreed strategic actions implemented on schedule; frequency of multisector performance reviews. |
| **Pillar 2 – A national laboratory network and multisector coordination platform** | Proportion of designated laboratories participating in the network; proportion of priority hazards with documented referral pathways; referral-to-result turnaround time for selected threats; multisector risk assessments/investigations. |
| **Pillar 3 – Quality systems, external quality assessment, and accreditation pathways** | EQA participation and satisfactory performance; proportion of priority laboratories meeting defined QMS milestones; progress of selected laboratories toward ISO 15189 or ISO/IEC 17025 accreditation |
| **Pillar 4 – Laboratory workforce capacity, competency development, and retention** | Proportion of staff in designated functions assessed against competency requirements; competency attainment; CPD completion; vacancy/retention measures for critical roles. |
| **Pillar 5 – Digital transformation, data governance, and One Health integration** | Electronic reporting coverage; completeness and timeliness of priority laboratory reporting; proportion of targeted LIS-surveillance interfaces meeting interoperability requirements; priority One Health hazards with routine cross-sector information exchange. |

Abbreviations: AMR, antimicrobial resistance; CPD, continuing professional development; CPHL, Central Public Health Laboratory; EQA, external quality assessment; HRH, human resources for health; IEC, International Electrotechnical Commission; JCDC, Jordan Center for Disease Control; LIS, laboratory information system; LQSI, Laboratory Quality Stepwise Implementation; MoH, Ministry of Health; QMS, quality management system; SOP, standard operating procedure; WHO, World Health Organization.
